# Controllable atom-photon entanglement via quantum interference near plasmonic nanostructure

**DOI:** 10.1038/s41598-021-04641-6

**Published:** 2022-01-13

**Authors:** Behzad Sangshekan, Mostafa Sahrai, Seyyed Hossein Asadpour, Jafar Poursamad Bonab

**Affiliations:** 1 Faculty of Physics, University of Tabriz, Tabriz, Iran; 2 Young Researchers and Elite Club, Central Tehran Branch, Islamic Azad University, Tehran, Iran; 3 Department of Optical and Laser Engineering, University of Bonab, Bonab, Iran

**Keywords:** Quantum information, Quantum optics

## Abstract

A five-level atomic system is proposed in vicinity of a two-dimensional (2D) plasmonic nanostructure with application in atom-photon entanglement. The behavior of the atom-photon entanglement is discussed with and without a control laser field. The amount of atom-photon entanglement is controlled by the quantum interference created by the plasmonic nanostructure. Thus, the degree of atom-photon entanglement is affected by the atomic distance from the plasmonic nanostructure. In the presence of a control field, maximum entanglement between the atom and its spontaneous emission field is observed.

## Introduction

The light-matter coherent interaction leads to an important phenomenon in quantum science such as quantum entanglement^1,2^. Quantum entanglement has widely been proposed due to its applications in quantum computing and quantum information technology^3,4^. Some important applications of entangled particles are their use in quantum algorithms^5^, quantum cryptography^6^, quantum networks^7,8^, and teleportation^9^. In last two decades, different approaches were presented to generate entangled particles^10,11^. Lately, matter-field entanglement has reached specific regard, because photons are used to carry the quantum information, and atoms are used to store it^12^. Many proposals were presented to produce the entanglement between quantum systems and their spontaneous emission field. Some of these articles are including the generation of entanglement between the atom and its spontaneous emission field via quantum entropy under the EIT conditions^13–15^. Time dependent behavior of the atom-photon entanglement is discussed when a four-level atom is embedded near the band edge of a photonic crystal^16^. The time evolution of the quantum entropy in a triple quantum dot molecule is controlled by the gate voltage and the rate of an incoherent pump field^17^. It was also shown that atom-photon entanglement can be controlled by the relative phase of the applied fields^18^, and the quantum interference parameter^19,20^. Basically, atom-photon entanglement can be achieved by the atomic coherence that is created by the coherent laser fields. Instead, when the system interacts with the surrounding reservoirs, due to the decoherence processes, the degree of entanglement and the information degrades. Spontaneous emission is an important phenomenon that leads to disentanglement of the two entangled states. However, spontaneous emission is an incoherent process, which can be controlled by placing the emitters in frequency-dependent reservoirs^21^, near the edges of photonic bandgaps (PBG)^22,23^, or in a microwave cavity^24^. The spectrum of the spontaneous emission strongly depends on the energy levels structure and the generated quantum coherence^25^. Therefore, due to quantum interference mechanism, the rate of spontaneous emission may be decreased or even suppressed.

On the other hand, the optical properties of the quantum emitters, i.e. atoms or semiconductor quantum dots, can significantly be modified when quantum systems are placed near the plasmonic nanostructures^26^. In vicinity of plasmonic nanostructures, the strong interaction between the electromagnetic field and the quantum emitters can be occurred^27^. Therefore, the optical response of the quantum emitter can be controlled using a hybrid quantum-plasmonic system. Quenching or enhancement of the spontaneous emission^28–30^, gain without population inversion^31^, enhancement of nonlinear optical response^32–34^ are described in hybrid plasmonic nanostructure. The effect of plasmonic nanostructure on optical grating^35^, probe field absorption^36^, and slow-light propagation^37^ was also proposed.

Now, we study the entanglement of a five-level quantum emitter coupled to a plasmonic nanostructure, namely a periodic 2D array of metal-coated dielectric nanospheres, and its spontaneous emission field. The combined density matrix approach and ab initio electromagnetic calculations are employed to discuss the response of the system. Steady-state population distribution of the various levels of the quantum emitter with and without the control laser field are investigated. We show that the population distribution and consequently atom-photon entanglement is strongly affected by the distance of quantum emitter from the plasmonic nanostructure. We show that the maximum value of entanglement can be achieved at a certain distance from the plasmonic nanostructure. We also prove that the degree of entanglement can effectively be controlled by the quantum interference between decay processes due to the proximity of the plasmonic nanostructure.

In the following discussion; we first present the coherently driven atomic model. Then, we obtain the relevant density matrix equations in the presence of the plasmonic nanostructure, and present the reduced entropy for calculating the atom-photon entanglement. In “Results and discussions” section some numerical results of the atom-photon entanglement are presented. Finally, the paper is concluded in “Conclusion” section.

## Model and equations

**Figure 1:**
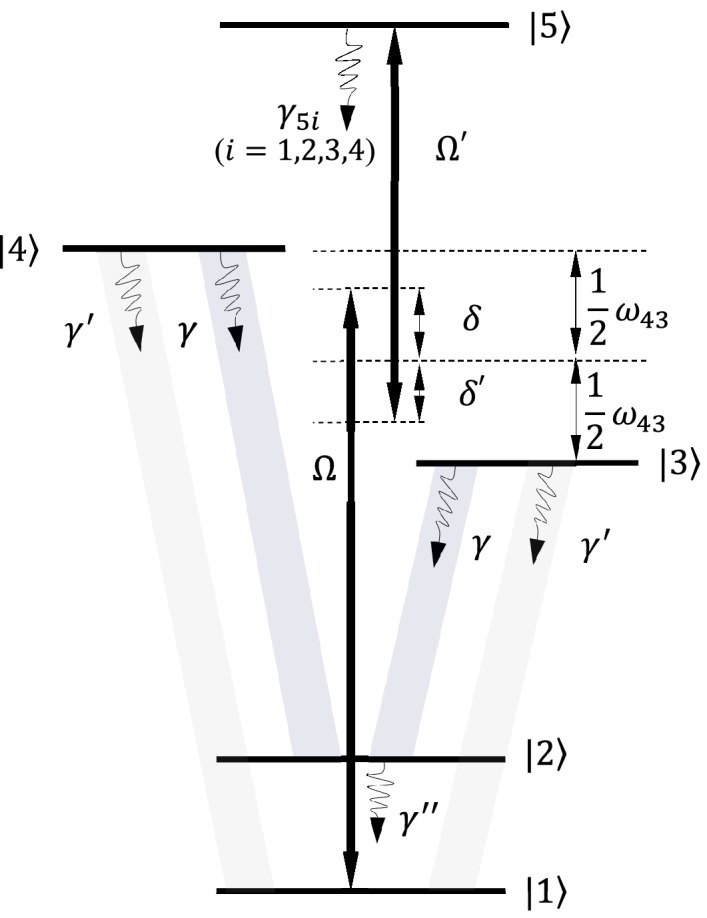
Energy diagram of a five-level atomic system.

**Figure 2:**
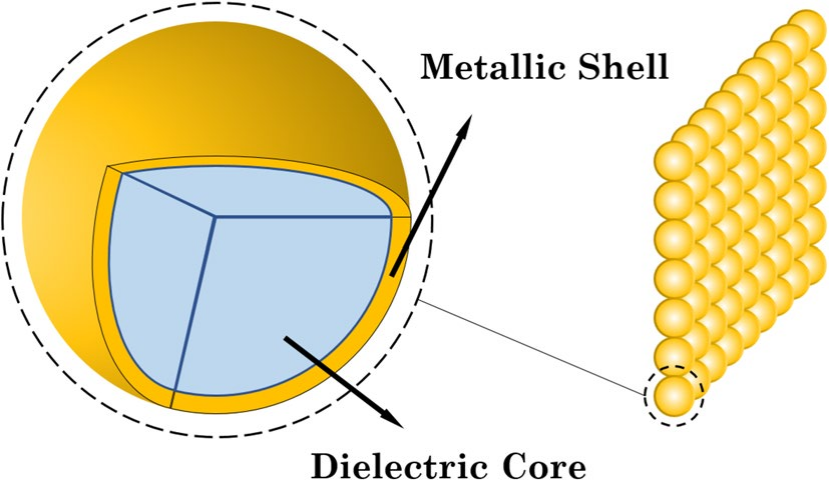
A 2D array of plasmonic nanostructures used in this study.

Consider a five-level atomic system with two lower levels $|1\rangle$ and $|2\rangle$, two closely lying middle Zeeman sublevels $|3\rangle$ and $|4\rangle$, and an additional higher-level $|5\rangle$ as depicted in Fig. 1. Assume this atomic system is fixed at a distance $d$ from the plasmonic nanostructure’s surface, which is located in vacuum space (Fig. 2). The strength of the interaction between the atom and nearly-resonant optical electric field $\widehat{\overrightarrow{E}}$ is characterized by the dipole moment operator $\widehat{\overrightarrow{\mu}}$. Hamiltonian for this interaction is ${\widehat{H}}_{int}=-\widehat{\overrightarrow{\mu }}.\widehat{\overrightarrow{E}}$. The diagonal matrix elements ${\overrightarrow{\mu }}_{ii}$ of this operator determine the dipole moments of the electron in the states $|i\rangle$, and are non-zero only in atoms with permanent dipole moments. The off-diagonal matrix elements ${\overrightarrow{\mu }}_{ij}$ are transition dipole moment, which demonstrates the transition of an electron from the state $|i\rangle$ to the state $|j\rangle$ and vice versa. The matrix elements ${\mu }_{ij}$ can be real or complex and ${\mu }_{ij}={\mu }_{ji}^{*}$. We take ${\overrightarrow{\mu }}_{32}={\overrightarrow{\mu }}_{42}=\overrightarrow{\mu }$, ${\overrightarrow{\mu }}_{31}={\overrightarrow{\mu }}_{41}={\overrightarrow{\mu }}{{^{\prime}}}$ and ${\overrightarrow{\mu }}_{53}={\overrightarrow{\mu }}_{54}={\overrightarrow{\mu }}{{^{\prime\prime}}}$. The diagonal matrix elements $\overrightarrow{\mu }$, ${\overrightarrow{\mu }}{{^{\prime}}}$ and ${\overrightarrow{\mu }}{{^{\prime\prime}}}$ are assumed to be real. The electric dipole moment operator is written as

1 $$\widehat{\overrightarrow{\mu }}={\overrightarrow{\mu }}{{^{\prime\prime}}}\left(|5\rangle \langle 3|{\widehat{\varepsilon }}_{-}+|5\rangle \langle 4|{\widehat{\varepsilon }}_{+}\right)+{\overrightarrow{\mu }}{{^{\prime}}}\left(|3\rangle \langle 1|{\widehat{\varepsilon }}_{-}+|4\rangle \langle 1|{\widehat{\varepsilon }}_{+}\right)+\overrightarrow{\mu }\left(|3\rangle \langle 2|{\widehat{\varepsilon }}_{-}+|4\rangle \langle 2|{\widehat{\varepsilon }}_{+}\right)+H.c.,$$

where ${\hat{\varepsilon }}_{+}$ and ${\hat{\varepsilon }}_{-}$ describe the right- and left-rotating unit vectors, which are defined as

2 $${\hat{\varepsilon }}_{\pm }=\left({\mathbf{e}}_{z}\pm i{\mathbf{e}}_{x}\right)/\sqrt{2}.$$

Here, ${\mathbf{e}}_{z}$ and ${\mathbf{e}}_{x}$ are unit vectors in $z$ and $x$ directions. Two linearly polarized continuous electric fields as $\overrightarrow{E}\left(t\right)={\mathbf{e}}_{z}{E}_{0}{\cos}\left(\nu t\right)$ and ${\overrightarrow{E}}^{{\prime}}\left(t\right)={\mathbf{e}}_{z}{E}_{0}^{{\prime}}{\cos}\left({\nu }^{{\prime}}t\right)$ are applied to the quantum system, where ${E}_{0}$ (${E}_{0}^{{\prime}}$) and $\nu$ (${\nu }^{{\prime}}$) are the amplitude and angular frequency of the electric field, respectively. The electric field $\overrightarrow{E}\left(t\right)$ drives transition $|1\rangle \leftrightarrow |i\rangle$ ($i=3, 4$), while controlling electric field ${\overrightarrow{E}}^{{\prime}}\left(t\right)$ couples level $|5\rangle$ to Zeeman sublevels $|3\rangle$ and $|4\rangle$. The atom–field interaction in dipole and rotating-wave approximation is described by Hamiltonian

3 $$H=-\frac{1}{2}\hslash\Omega {e}^{-i\left(\delta +\frac{1}{2}{\omega }_{43}\right)t}|3\rangle \langle 1|-\frac{1}{2}\hslash\Omega {e}^{-i\left(\delta -\frac{1}{2}{\omega }_{43}\right)t}|4\rangle \langle 1|-\frac{1}{2}\hslash {\Omega }^{{\prime}}{e}^{-i\left({\delta }^{{\prime}}-\frac{1}{2}{\omega }_{43}\right)t}|5\rangle \langle 3|-\frac{1}{2}\hslash {\Omega }^{{\prime}}{e}^{-i\left({\delta }^{{\prime}}+\frac{1}{2}{\omega }_{43}\right)t}|5\rangle \langle 4|\,+\, H.c..$$

Here, $\delta$ (${\delta }^{{\prime}})$ is the detuning between respected energy levels and applied fields, which is measured from average transition frequencies of level $|3\rangle$ and level $|4\rangle$ with level $|1\rangle$ ($|5\rangle$). So, detunings define as $\delta =\nu -\left({\omega }_{31}+\frac{1}{2}{\omega }_{43}\right)=\nu -\left({\omega }_{41}-\frac{1}{2}{\omega }_{43}\right)$, and ${\delta }^{{\prime}}={\nu }^{{\prime}}-\left({\omega }_{54}+\frac{1}{2}{\omega }_{43}\right)={\nu }^{{\prime}}-\left({\omega }_{53}-\frac{1}{2}{\omega }_{43}\right)$, where, ${\omega }_{ij}$ are transition frequencies between the energy level $|i\rangle$ and level $|j\rangle$. The parameters $\Omega =\mu {E}_{0}/\sqrt{2}\hslash$ and ${\Omega }^{{\prime}}={\mu }^{{{\prime}}{{\prime}}}{E}_{0}^{{\prime}}/\sqrt{2}\hslash$ are the corresponding Rabi-frequencies.

The spontaneous emission rates from the excited level $|5\rangle$ to the other lower levels are denoted by $2{\gamma }_{5i}$ ($i=1, 2, 3, 4$). The transitions from level $|5\rangle$ to level $|3\rangle$ and level $|4\rangle$ are influenced by the interaction of the quantum system with free-space vacuum modes, so these transitions are not affected by the plasmonic nanostructure. The Zeeman sublevels $|3\rangle$ and $|4\rangle$ spontaneously decay to level $|1\rangle$ ($|2\rangle$) with decay rates $2{\gamma }_{3}^{{\prime}}$ ($2{\gamma }_{3}$) and $2{\gamma }_{4}^{{\prime}}$ ($2{\gamma }_{4}$), respectively. Moreover, we assume the transitions from level $|3\rangle$ and level $|4\rangle$ to level $|2\rangle$ spectrally be located in the surface-plasmon band of the plasmonic nanostructure, while the transitions from level $|3\rangle$ and level $|4\rangle$ to level $|1\rangle$ are also far from the surface-plasmon bands.

Note that, if we consider the transitions from level $|3\rangle$ and level $|4\rangle$ to level $|1\rangle$ near the surface plasmon band of the plasmonic nanostructure, the Rabi frequency of the coupling light, which couples the ground level $|1\rangle$ to upper-intermediate states, may be affected by plasmonic nanostructure. In fact, the plasmonic nanostructure affects the Rabi frequency of coupling light, and its value may be changed.

For simplicity, we assume ${\omega }_{43}$ to be relatively small that equals to a few ${\Gamma }_{0}$ (decay rate of level $|3\rangle$ and level $|4\rangle$ to level $|2\rangle$ in the vacuum). Later, the energy of both middle levels is taken to be the same; thus decay rates from level $|3\rangle$ and level $|4\rangle$ to level $|1\rangle$ are coupled by the same vacuum mods. Therefore, these transitions are free-space spontaneous decay. In addition, spontaneous decay from level $|3\rangle$ and level $|4\rangle$ to level $|2\rangle$ are coupled by the same mods that affected by plasmonic nanostructure. Then, we can assume ${\gamma }_{3}={\gamma }_{4}=\gamma$ and ${\gamma }_{3}^{{\prime}}={\gamma }_{4}^{{\prime}}={\gamma }^{{\prime}}$^38^.

Considering the Hamiltonian described in Eq. (3), the density matrix equations of motion in rotating frame are obtained by quantum Liouville equation

4 $$\dot{\rho }=-\frac{i}{\mathrm{\hslash }}\left[H,\rho \right]+\mathcal{L}\rho ,$$

where $\mathcal{L}\rho$ is Liouville operator and expresses the dissipation processes which is given by

5 $$\begin{aligned}\mathcal{L}\rho &=-\gamma \left[{\sigma }_{32}{\sigma }_{23}\rho -2{\sigma }_{23}\rho {\sigma }_{32}+\rho {\sigma }_{32}{\sigma }_{23}\right]-\gamma \left[{\sigma }_{42}{\sigma }_{24}\rho -2{\sigma }_{24}\rho {\sigma }_{42}+\rho {\sigma }_{42}{\sigma }_{24}\right] \\ & \quad -{\gamma }^{{\prime}}\left[{\sigma }_{31}{\sigma }_{13}\rho -2{\sigma }_{13}\rho {\sigma }_{31}+\rho {\sigma }_{31}{\sigma }_{13}\right]-{\gamma }^{{\prime}}\left[{\sigma }_{41}{\sigma }_{14}\rho -2{\sigma }_{14}\rho {\sigma }_{41}+\rho {\sigma }_{41}{\sigma }_{14}\right] \\ & \quad -{\gamma }^{{{\prime}}{{\prime}}}\left[{\sigma }_{21}{\sigma }_{12}\rho -2{\sigma }_{12}\rho {\sigma }_{21}+\rho {\sigma }_{21}{\sigma }_{12}\right]-{\gamma }_{51}\left[{\sigma }_{51}{\sigma }_{15}\rho -2{\sigma }_{15}\rho {\sigma }_{51}+\rho {\sigma }_{51}{\sigma }_{15}\right] \\ & \quad -{\gamma }_{52}\left[{\sigma }_{52}{\sigma }_{25}\rho -2{\sigma }_{25}\rho {\sigma }_{52}+\rho {\sigma }_{52}{\sigma }_{25}\right]-{\gamma }_{53}\left[{\sigma }_{53}{\sigma }_{35}\rho -2{\sigma }_{35}\rho {\sigma }_{53}+\rho {\sigma }_{53}{\sigma }_{35}\right] \\ & \quad -{\gamma }_{54}\left[{\sigma }_{54}{\sigma }_{45}\rho -2{\sigma }_{45}\rho {\sigma }_{54}+\rho {\sigma }_{54}{\sigma }_{45}\right]-\kappa {e}^{-i{\omega }_{43}t}\left[{\sigma }_{32}{\sigma }_{24}\rho -2{\sigma }_{24}\rho {\sigma }_{32}+\rho {\sigma }_{32}{\sigma }_{24}\right] \\ & \quad -\kappa {e}^{i{\omega }_{43}t}\left[{\sigma }_{42}{\sigma }_{23}\rho -2{\sigma }_{23}\rho {\sigma }_{42}+\rho {\sigma }_{42}{\sigma }_{23}\right],\end{aligned}$$

where, ${\sigma }_{ij}=|i\rangle \langle j|$ is the atom transition operator (see Eq. 1 in Supplementary Note). $\kappa$ represents the coupling coefficient between level $|3\rangle$ and level $|4\rangle$. This coefficient is due to anisotropic vacuum influence on spontaneous emission due to the existence of plasmonic nanostructure (anisotropic Purcell effect)^39^, which arises due to the quantum interference mechanism^40^. The values of $\gamma$ and $\kappa$ can be obtained by the dyadic electromagnetic Green’s tensor $\mathbf{G}\left(\overrightarrow{r},\overrightarrow{r};\omega \right)$^41^, as

6a $$\gamma =\frac{{\mu }_{0}{\mu }^{2}{\omega }^{2}}{2\hslash }{\widehat{\varepsilon }}_{-}.Im\left[\mathbf{G}\left(\overrightarrow{r},\overrightarrow{r};\omega \right)\right].{\widehat{\varepsilon }}_{+,}$$

6b $$\kappa =\frac{{\mu }_{0}{\mu }^{2}{\omega }^{2}}{2\hslash }{\widehat{\varepsilon }}_{+}.Im\left[\mathbf{G}\left(\overrightarrow{r},\overrightarrow{r};\omega \right)\right].{\widehat{\varepsilon }}_{+},$$

where $\omega =\left({\omega }_{4}+{\omega }_{3}\right)/2-{\omega }_{2}$, $\overrightarrow{r}$ displays the position of the atomic system, and ${\mu }_{0}$ refers to the permeability of vacuum space. Due to the Eq. (5), we can write the values of $\gamma$ and $\kappa$^42^, as

7a $$\gamma =\frac{{\mu }_{0}{\mu }^{2}{\omega }^{2}}{2\hslash }Im\left[{G}_{\perp }\left(\overrightarrow{r},\overrightarrow{r};\omega \right)+{G}_{\parallel }\left(\overrightarrow{r},\overrightarrow{r};\omega \right)\right]=\frac{1}{2}\left({\Gamma }_{\perp }+{\Gamma }_{\parallel }\right),$$

7b $$\kappa =\frac{{\mu }_{0}{\mu }^{2}{\omega }^{2}}{2\hslash }Im\left[{G}_{\perp }\left(\overrightarrow{r},\overrightarrow{r};\omega \right)-{G}_{\parallel }\left(\overrightarrow{r},\overrightarrow{r};\omega \right)\right]=\frac{1}{2}\left({\Gamma }_{\perp }-{\Gamma }_{\parallel }\right).$$

Moreover, ${G}_{\perp }\left(\overrightarrow{r},\overrightarrow{r};\omega \right)={G}_{zz}\left(\overrightarrow{r},\overrightarrow{r};\omega \right)$, ${G}_{\parallel }\left(\overrightarrow{r},\overrightarrow{r};\omega \right)={G}_{xx}\left(\overrightarrow{r},\overrightarrow{r};\omega \right)$ indicates the elements of the electromagnetic wave Green’s tensor. Here, index $\parallel$ ($\perp$) denotes the dipole oriented parallel (normal) along the x-axis (z-axis) to the surface of the plasmonic nanostructure^38^. Therefore, we express the spontaneous emission rates in parallel and normal directions to the surface of the plasmonic nanostructure as

8a $${\Gamma }_{\parallel }={\mu }_{0}{\mu }^{2}{\omega }^{2}Im\left[{G}_{\parallel }\left(\overrightarrow{r},\overrightarrow{r};\omega \right)\right]/\hslash ,$$

8b $${\Gamma }_{\perp }={\mu }_{0}{\mu }^{2}{\omega }^{2}Im\left[{G}_{\perp }\left(\overrightarrow{r},\overrightarrow{r};\omega \right)\right]/\hslash .$$

Now, we introduce the quantum interference parameter as $p=\frac{{\Gamma }_{\perp }-{\Gamma }_{\parallel }}{{\Gamma }_{\perp }+{\Gamma }_{\parallel }}=\frac{\kappa }{\gamma }$ that arises due to existence of plasmonic nanostructure. Spontaneous emission may be enhanced or even quenched via the quantum interfere mechanism depending on ${\Gamma }_{\parallel }$ and ${\Gamma }_{\perp }$. When quantum system is very far from the plasmonic nanostructure, i.e. ${\Gamma }_{\perp }={\Gamma }_{\parallel }$ and $\kappa =0$, no quantum interference appears^37,43,44^. However, if the emitter is placed near the plasmonic nanostructure, i.e. ${\Gamma }_{\parallel }=0$, the parameter $\kappa$ is identical and quantum interference is maximum.

Here, we propose a 2D array of plasmonic nanostructures, where metal-coated silica nanospheres are connected to each other (Fig. 2). The shell has a frequency-dependent dielectric function represented by a Drude-model electric permittivity

9 $$\epsilon \left(\omega \right)=1-\frac{{\omega }_{p}^{2}}{\omega \left(\omega +i/\tau \right)},$$

where $\tau$ demonstrates the relaxation time for electrons of metal conduction-band, and ${\omega }_{p}$ represents the plasma frequency of the bulk. The plasma frequency for silver metal is $\hslash {\omega }_{p}= 3.8 eV$. Also, this value specifies the length order of the system as $c/{\omega }_{p}\approx 22 \; \text{nm}$. For $Si{O}_{2}$ the dielectric constant is $\epsilon =2.1$. In the calculation process, we assume ${\tau }^{-1}=0.1 {\omega }_{p}$. This square lattice has a lattice constant $a=104\; \text{nm}$ and radius of the sphere (core) $S=52\; \text{nm}$ (${S}_{c}=36.4\; \text{nm}$)^27^.

Now, we consider a model with two subsystems such as atom ($A$) and its spontaneous emission photon ($F$). If this atom–field pure state system cannot be expressed as a tensor product of the two subsystems ($\rho \ne {\rho }_{A}\otimes {\rho }_{F}$), the atom and its spontaneous emission photon will be entangled. We utilize the reduced quantum entropy to measure the amount of atom-photon entanglement. To measure the degree of entanglement of a pure state $\rho$, we only need the atomic quantum entropy ${S}_{A}\left(t\right)$^45,46^. The reduced quantum entropy for the bipartite pure system is the von-Neumann reduced entropy as defined

10 $${S}_{A\left(F\right)}\left(t\right)=-Tr\left[{\rho }_{A\left(F\right)}{{\log}}_{2}{\rho }_{A\left(F\right)}\right].$$

We can also represent the atomic quantum entropy according to terms eigenvalues ${\lambda }_{A\left(F\right)}\left(t\right)$ of reduced density operators as a degree of entanglement ($DEM$)

11 $$DEM={S}_{A}\left(t\right)={S}_{F}\left(t\right)=-\sum_{j=1}^{5}{\lambda }_{A}^{\left(j\right)}\left(t\right){{\log}}_{2}{\lambda }_{A}^{\left(j\right)}\left(t\right),$$

where ${\lambda }_{A}^{\left(j\right)}$ are the eigenvalues of the ${\rho }_{A}$. To achieve a quantum pure state, we assume all the atoms initially in their ground states (${\rho }_{11}=1$). If this reduced density matrix, ${S}_{A}\left(t\right)$, describes a (maximally) mixed subsystem, then the whole pure state $\rho$, will be (maximally) entangled. When entropy of entanglement is equal to $E\left(\rho \right)={{\log}}_{2}\left[\text{min}\left({d}_{A},{d}_{F}\right)\right]$, we will have a maximally entangled state. In addition, the amount of the entropy is limited by the $0\le S\left(\rho \right)\le {{\log}}_{2}D$, where $D$ is the dimension of a Hilbert space $H$. The entropy is maximized when the quantum state is maximally mixed, i.e. $\rho =\frac{1}{D}I$, where $I$ is an identity matrix^47^. Therefore, by evenly distributed population on the atomic levels, we will have the maximum amount of entanglement. Hence, the rate of spontaneous emission affects the population distribution leading to change of the atom-photon entanglement.

## Results and discussions

Now, density matrix equations (4) along with Eq. (11) should numerically be solved to reach the $DEM$. In this regard, $DEM$ relates to the atomic parameters given in supplementary Eq. (1), and will characterize the degree of atom-photon entanglement. In following discussion, all the parameters are scaled by the parameter ${\Gamma }_{0}$ that is the decay rate of spontaneous emission in free space. The decay rates from level $|5\rangle$ to level $|i\rangle$ ($i=1, 2, 3, 4$) are defiend as ${\gamma }_{51}={\gamma }_{52}=0.02{\Gamma }_{0}$ and ${\gamma }_{53}={\gamma }_{54}={\Gamma }_{0}$. The transitions from level $|3\rangle$ and level $|4\rangle$ to level $|1\rangle$ and from level $|2\rangle$ to level $|1\rangle$ are the dipole-allowed spontaneous decay rates that are equal to ${\gamma }^{{\prime}}={\Gamma }_{0}$ and ${\gamma }^{{{\prime}}{{\prime}}}=0.2 {\Gamma }_{0}$, respectively. For transitions from level $|3\rangle$ and level $|4\rangle$ to level $|2\rangle$, the dipole-allowed spontaneous decay rates are equal to $\gamma$. The parameters $\gamma$ and $\kappa$ are obtained according to Eq. (8) in terms of ${\Gamma }_{\perp }$ and ${\Gamma }_{\parallel }$ for the distances expressed in Table 1. Note that the values for the controlling parameters are chosen according to the ^87^Rb atom as a areal atomic system. In fact, the proposed five-level quantum system can be realized in hyperfine sublevels of ^87^Rb. Thus the proposed levels are labeled by the spectroscopic definition as $|1\rangle =|5{S}_{1/2};F=1, {m}_{F}=0\rangle$, $|2\rangle =|5{S}_{1/2};F=2, {m}_{F}=0\rangle$, $|3\rangle =|5{P}_{1/2};{F}^{{\prime}}=2, {m}_{{F}^{{\prime}}}=-1\rangle$, $|4\rangle =|5{P}_{1/2};{F}^{{\prime}}=2, {m}_{{F}^{{\prime}}}=+1\rangle$, and $|5\rangle =|6{S}_{1/2};{F}^{{{\prime}}{{\prime}}}=2, {m}_{{F}^{{{\prime}}{{\prime}}}}=0\rangle$. In this regards transition $\left|1\right.\rangle \to |2\rangle$ is corresponding to the ${D}_{1}$ line of ^87^Rb^48–51^.

**Table 1:** The values of ${\Gamma }_{\perp }$ and ${\Gamma }_{\parallel }$ according to distances of the atom from the plasmonic nanostructure for $\hslash \omega =2.4 eV$ (${\Gamma }_{0}=2\pi \times 2.9 \, \text{MHz}$).

Distance $d \; ( \text{nm})$	10.4	20.8	31.2	41.6	52	∞
${\Gamma }_{\perp }$ (${\Gamma }_{0})$	27.081	6.417	1.774	0.559	0.196	1
${\Gamma }_{\parallel }$ (${\Gamma }_{0})$	0.105	0.038	0.021	0.021	0.026	1
$\gamma \; ({\Gamma }_{0})$	13.958	3.228	0.898	0.290	0.111	1
$\kappa \; ({\Gamma }_{0})$	13.853	3.190	0.877	0.269	0.085	0
$p$	0.993	0.988	0.977	0.928	0.766	0

Here, we are interested in studying the steady-state and dynamical behavior of the atom-photon entanglement under the condition $\delta =0$. Note that maximum value of the entanglement for the N-levels atomic system is ${DEM}_{max}={{\log}}_{2}N$, where we use the concept of normalized entanglement as a ratio of $DEM\left(x\right)$ per ${DEM}_{max}$. Here, $x$ represents variables such as Rabi-frequencies and quantum interference that may change the $DEM$. Hence, the amount of normalized entanglement of any N-level atomic system can be expressed as $0\le \left(DEM\left(x\right)/{DEM}_{max}\right)\le 1$. For the proposed five-level quantum system, the maximum value of expected entanglement must be ${DEM}_{max}={{\log}}_{2}5=2.32$, where its normalized value is $0\le \left\{DEM\left(x\right)/2.32\right\}\le 1$.

**Figure 3:**
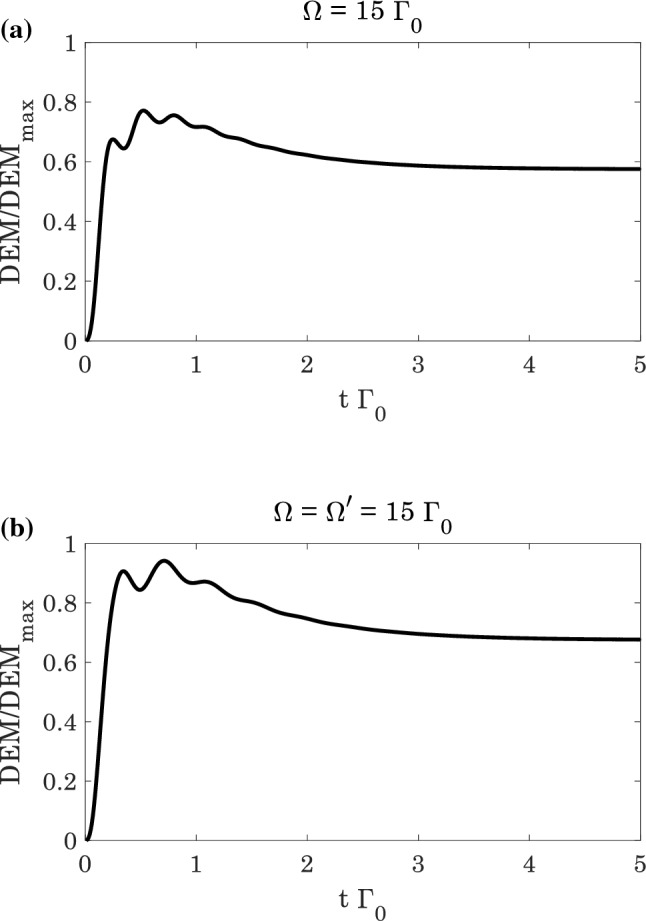
The time evolution of normalized $DEM$ ($\left(DEM\left(t\right)/{DEM}_{max}\right)\le 1$) of the quantum system in the absence of plasmonic nanostructure for (**a**) ${\Omega }^{{{\prime}}}/{\Gamma }_{0}=0$, and (**b**) ${\Omega }^{{{\prime}}}/{\Gamma }_{0}=\Omega /{\Gamma }_{0}=15$.

Figure 3, displays the time-dependent normalized behavior of $DEM$ in the absence of plasmonic nanostructure, i.e. $d\to \infty$, where ${\Gamma }_{\perp }={\Gamma }_{\parallel }$ and $\kappa =0$. The atoms initially are in their ground state, ${\rho }_{11}=1$ and ${\rho }_{ij}=0$, thus the whole system is in a pure state. Therefore, the atom and its spontaneous emission field is initially disentangled. By increasing the normalized time, the two subsystems including the atom and photon reache to a mixed state, and the $DEM$ increases by the time. In the absence of the control field, i.e. ${\Omega }^{{{\prime}}}=0$, the five-level atomic system converts to a four-level one. Without the control field (Fig. 3a), the $DEM$ reaches to $0.56$, while it increases to $0.68$ for ${\Omega }^{{{\prime}}}\ne 0$ (Fig. 3b). So, in the presence of control field, the $DEM$ is higher than the case without control field. This is due to the existence of spontaneous emission from upper level $|5\rangle$ to lover levels leading to equally population distribution of each levels.

**Figure 4:**
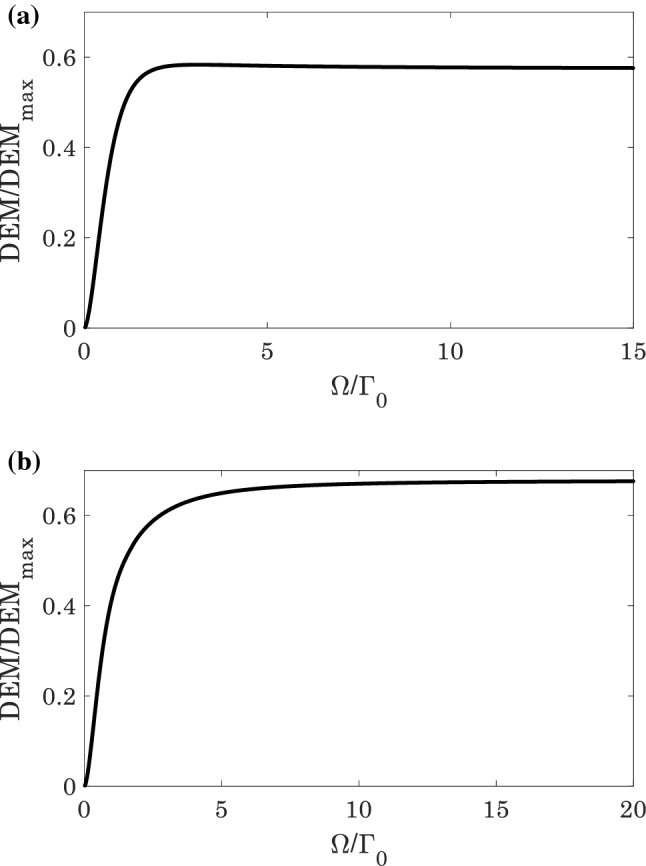
The normalized $DEM$ ($\left(DEM\left(\Omega \right)/{DEM}_{max}\right)\le 1$) of the quantum system in the absence of plasmonic nanostructure for (**a**) ${\Omega }^{{{\prime}}}/{\Gamma }_{0}=0$, and (**b**) ${\Omega }^{{{\prime}}}/{\Gamma }_{0}=\Omega /{\Gamma }_{0}$.

The steady-state behavior of the normalized $DEM$ as a function of the Rabi-frequency $\Omega /{\Gamma }_{0}$ without plasmonic nanostructure is displayed in Fig. 4. The results are in a good agreement with Fig. 3.

**Figure 5:**
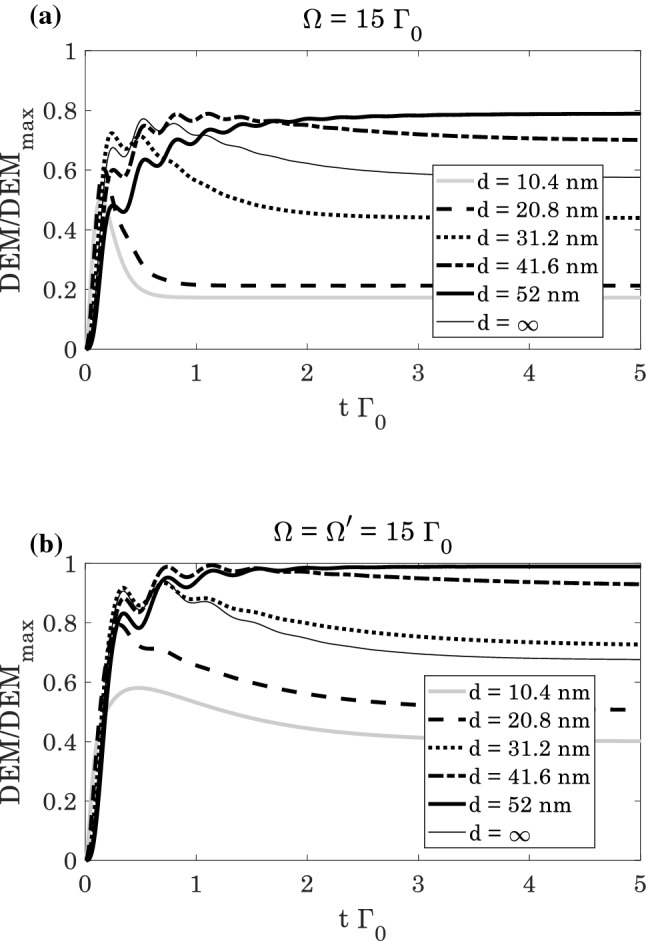
The time evolution of normalized $DEM$ ($\left(DEM\left(t\right)/{DEM}_{max}\right)\le 1$) of the quantum system in the presence of plasmonic nanostructure for (**a**) ${\Omega }^{{{\prime}}}/{\Gamma }_{0}=0$, and (**b**) ${\Omega }^{{{\prime}}}/{\Gamma }_{0}=\Omega /{\Gamma }_{0}=15$.

In Fig. 5, the time evolution of normalized $DEM$ is presented in the presence of plasmonic nanostructure with and without control field. Similarly, the atom and its spontaneous emission field are initially disentangled, but by increasing the normalized time the $DEM$ also increases. In vicinity of nanostructure, the atom and its spontaneous emission field undergoes different degrees of entanglement depending on the distance of atom from the plasmonic nanostructure. We find that for both cases ${\Omega }^{{\prime}}=0$ and ${\Omega }^{{\prime}}\ne 0$, by increasing the distance of atom from the plasmonic nanostructure, the atom-photon entanglement increases (Fig. 5). Similar to Fig. 3, for ${\Omega }^{{\prime}}\ne 0$ the $DEM$ is higher than ${\Omega }^{{\prime}}=0$. But in $d=52 \; \text{nm}$ the amount of $DEM$ for ${\Omega }^{{\prime}}=0$ is about $0.8$, while it reaches to $1$ for ${\Omega }^{{\prime}}\ne 0$. This $DEM$ is the optimal normalized entanglement. Note that the quantum interference arising from the existence of plasmonic nanostructure has crucial role in atom-photon entanglement. By increasing the distance of the emitter from the plasmonic nanostructure, quantum interference reduces as can be seen from table (1). Thus, the spontaneous emission from level $|3\rangle$ and level $|4\rangle$ to level $|2\rangle$ can be controlled by the quantum interference that depends on the distance of atom from the nanostructure. Then, $DEM$ will change just by the spontaneous emission of level $|3\rangle$ and level $|4\rangle$ to level $|2\rangle$, where it controls by the quantum interference.

**Figure 6:**
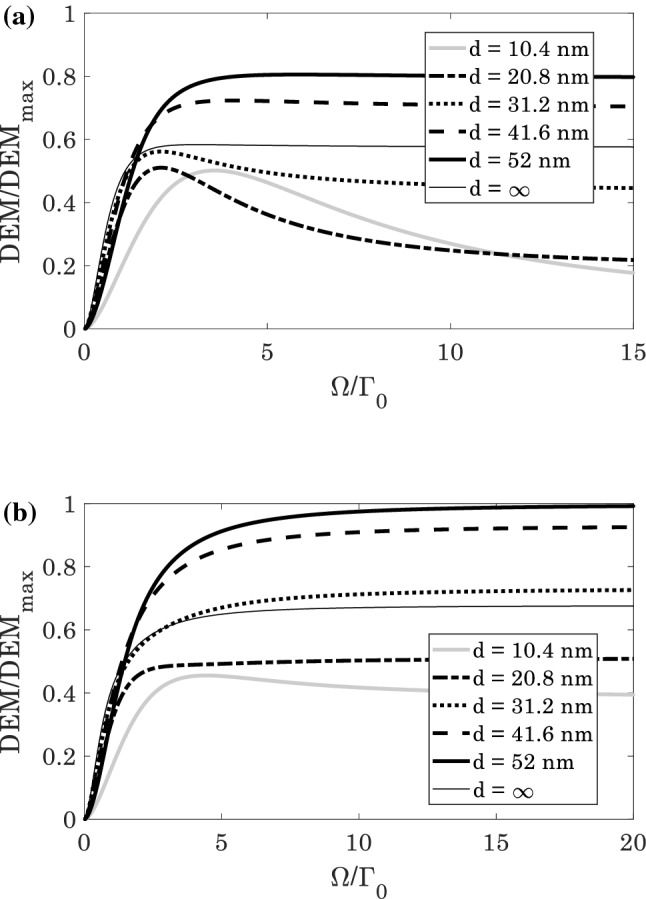
The normalized $DEM$ ($\left(DEM\left(\Omega \right)/{DEM}_{max}\right)\le 1$) of the quantum system in the presence of plasmonic nanostructure for (**a**) ${\Omega }^{{{\prime}}}/{\Gamma }_{0}=0$, and (**b**) ${\Omega }^{{{\prime}}}/{\Gamma }_{0}=\Omega /{\Gamma }_{0}$.

The normalized $DEM$ as a function of the Rabi-frequency $\Omega /{\Gamma }_{0}$ for various distances is denoted in Fig. 6. It is obviously realized that by increasing the distance of atom from the plasmonic nanostructure, the atom-photon entanglement increases. These results are confirmed by Fig. 5, and we concluded that the amount of atom-photon entanglement in vicinity of the plasmonic nanostructure can be controlled just by the distance $d$.

In order to discuss the physical mechanism of the obtained results, the population distribution of the bare and dressed states is analyzed in the following discussion.

**Figure 7:**
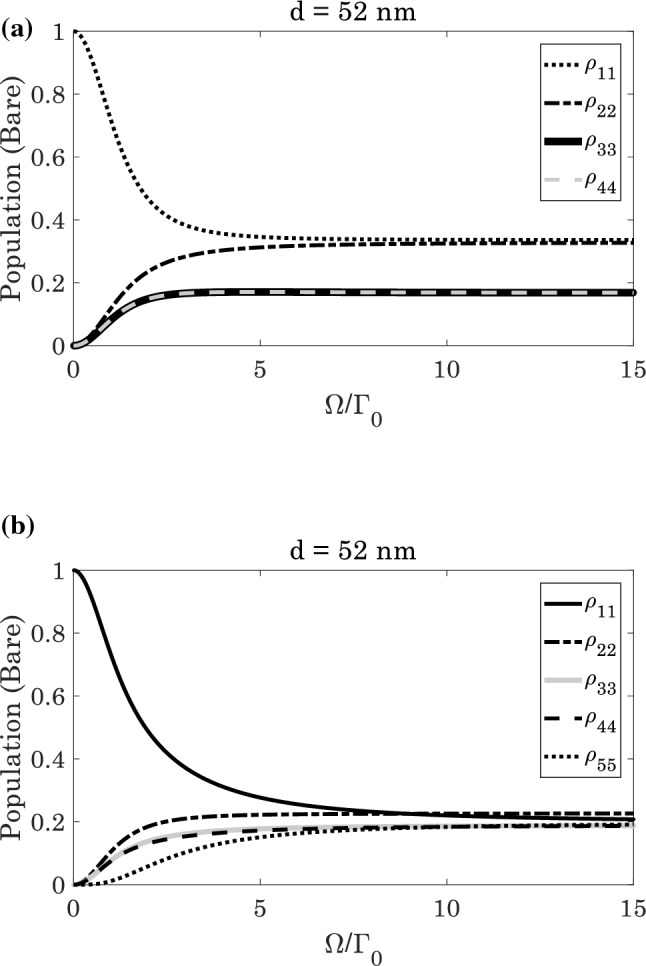
The population distribution of the bare states as a function of Rabi-frequencies at $d=52 \; \text{nm}$ for (**a**) ${\Omega }^{{{\prime}}}/{\Gamma }_{0}=0$, and (**b**) ${\Omega }^{{{\prime}}}/{\Gamma }_{0}=\Omega /{\Gamma }_{0}$.

Figure 7 shows the population distribution of the bare states. We observe that the population is not equally distributed among the bare states, and this may reduce the $DEM$ (Fig. 7a). However, when the population is equally distributed among the bare states, the maximum atom-photon entanglement is observed. Thus the obtained results in previous figures are approved by the population distribution.

To give more physical insight on the maximal atom-photon entanglement, the dressed state formalism is also presented. Without the control field, i.e. ${\Omega }^{{{\prime}}}=0$, the transformed Hamiltonian can be written as

12 $$\tilde{H }=-\hslash \left(\delta +\frac{1}{2}{\omega }_{43}\right)|3\rangle \langle 3|-\hslash \left(\delta -\frac{1}{2}{\omega }_{43}\right)|4\rangle \langle 4|-\left\{\frac{1}{2}\hslash\Omega \left[|3\rangle \langle 1|+|4\rangle \langle 1|\right]+H.c.\right\}.$$

By calculating the eigenvalues of this Hamiltonian, using the relation $\mathrm{det}\left(\tilde{H }-\lambda I\right)=0$, we obtain

13 $${\lambda }^{3}+2\hslash \delta {\lambda }^{2}+{\hslash }^{2}\left[{\delta }^{2}-\frac{1}{4}{\omega }_{43}^{2}-\frac{1}{2}{\left|\Omega \right|}^{2}\right]\lambda -\frac{1}{2}{\hslash }^{3}{\left|\Omega \right|}^{2}\delta =0,$$

where $\lambda$’s are eigenvalues of this Hamiltonian operator. For $\delta =0$, the eigenvalue $\lambda$’s are given by

14 $${\lambda }_{{1,2},3}=0,\pm \frac{1}{2}\hslash {\Omega }_{d},$$

where ${\Omega }_{d}\equiv \sqrt{{\omega }_{43}^{2}+2{\left|\Omega \right|}^{2}}$ called generalized Rabi-frequency. So, the corresponding dressed states are

15 $$\begin{aligned} |\alpha \rangle &=\frac{\left|\Omega \right|}{{\Omega }_{d}}\left(\frac{{\omega }_{43}}{\Omega }|1\rangle -|3\rangle +|4\rangle \right), \\ |2\rangle &=|2\rangle , \\ |\beta \rangle & =\frac{{\left|\Omega \right|}^{2}}{{\Omega }_{d}^{2}+{\omega }_{43}{\Omega }_{d}}\left(\frac{{\omega }_{43}+{\Omega }_{d}}{\Omega }|1\rangle +\frac{{\omega }_{43}^{2}+{\left|\Omega \right|}^{2}+{\omega }_{43}{\Omega }_{d}}{{\left|\Omega \right|}^{2}}|3\rangle +|4\rangle \right), \\ |\eta \rangle & =\frac{{\left|\Omega \right|}^{2}}{{\Omega }_{d}^{2}-{\omega }_{43}{\Omega }_{d}}\left(\frac{{\omega }_{43}-{\Omega }_{d}}{\Omega }|1\rangle +\frac{{\omega }_{43}^{2}+{\left|\Omega \right|}^{2}-{\omega }_{43}{\Omega }_{d}}{{\left|\Omega \right|}^{2}}|3\rangle +|4\rangle \right). \end{aligned}$$

When the control field ${\Omega }^{{{\prime}}}$ is on, transformed Hamiltonian are written as

16 $$\tilde{H }=-\hslash \left(\delta +\frac{1}{2}{\omega }_{43}\right)|3\rangle \langle 3|-\hslash \left(\delta -\frac{1}{2}{\omega }_{43}\right)|4\rangle \langle 4|-\hslash \left(\delta +{\delta }^{{\prime}}\right)|5\rangle \langle 5|-\frac{1}{2}\hslash \left\{\Omega \left(|3\rangle \langle 1|+|4\rangle \langle 1|\right)+{\Omega }^{{{\prime}}}\left(|5\rangle \langle 3|+|5\rangle \langle 4|\right)+H.c.\right\}.$$

Thus, using the relation $\text{det}\left(\tilde{H }-\lambda I\right)=0$, we can reach to

17 $$\left[\lambda +\hslash \left(\delta +{\delta }^{{\prime}}\right)\right]\left[4\lambda {\left(\lambda +\hslash \delta \right)}^{2}-\lambda {\hslash }^{2}{\omega }_{43}^{2}-2{\hslash }^{2}\left(\lambda +\hslash \delta \right){\left|{\Omega }_{1}\right|}^{2}\right]-2\lambda {\hslash }^{2}\left(\lambda +\hslash \delta \right){\left|{\Omega }_{2}\right|}^{2}=0.$$

For $\delta ={\delta }^{{\prime}}=0$, eigenvalues $\lambda$’s are obtained as follows

18 $${\lambda }_{{1,2},{3,4}}={0,0},\pm \frac{1}{2}\hslash {\Omega }_{d},$$

where, generalized Rabi-frequency is ${\Omega }_{d}\equiv \sqrt{{\omega }_{43}^{2}+2\left({\left|{\Omega }_{1}\right|}^{2}+{\left|{\Omega }_{2}\right|}^{2}\right)}$. So, the corresponding dressed states are

19 $$\begin{aligned} |\alpha \rangle & =-\frac{1}{\sqrt{2}}\left(-\frac{{\Omega }^{*}}{\Omega }|1\rangle -|5\rangle \right), \\ |2\rangle & =|2\rangle , \\ |\beta \rangle & =\frac{\left|\Omega \right|}{\sqrt{{\omega }_{43}^{2}+2{\left|\Omega \right|}^{2}}}\left(\frac{{\omega }_{43}}{\Omega }|1\rangle -|3\rangle +|4\rangle \right), \\ |\eta \rangle & =\frac{\left|\Omega \right|}{{\Omega }_{d}}\left(\frac{{\Omega }^{*}}{\Omega }|1\rangle +\frac{{\omega }_{43}+{\Omega }_{d}}{2\Omega }|3\rangle -\frac{{\omega }_{43}-{\Omega }_{d}}{2\Omega }|4\rangle +|5\rangle \right), \\ |\xi \rangle & =\frac{\left|\Omega \right|}{{\Omega }_{d}}\left(\frac{{\Omega }^{*}}{\Omega }|1\rangle +\frac{{\omega }_{43}-{\Omega }_{d}}{2\Omega }|3\rangle -\frac{{\omega }_{43}+{\Omega }_{d}}{2\Omega }|4\rangle +|5\rangle \right). \end{aligned}$$

**Figure 8:**
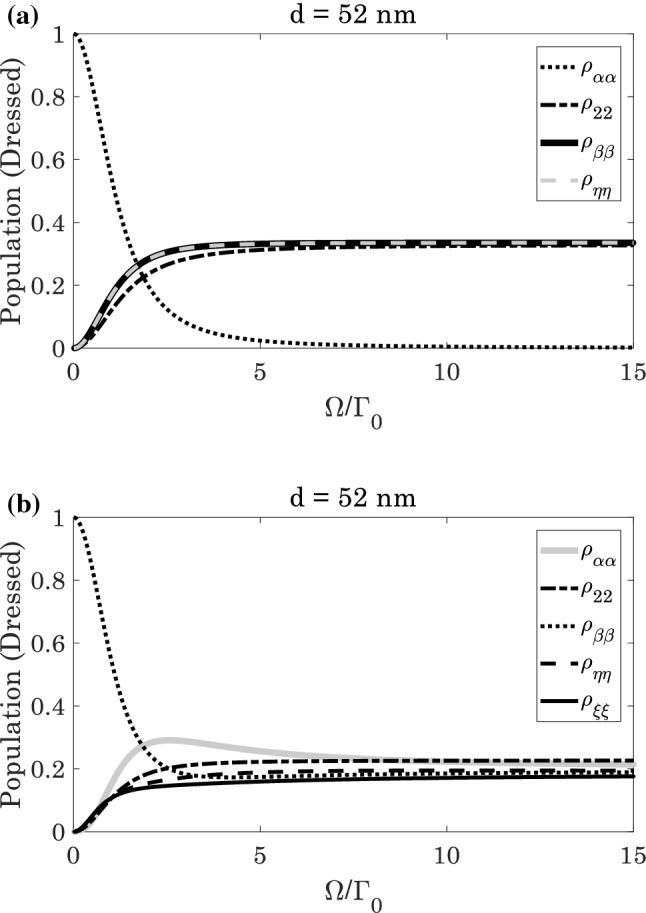
The dressed state population distribution as a function of Rabi-frequencies at distance $d=52 \; \text{nm}$ for (**a**) ${\Omega }^{{{\prime}}}/{\Gamma }_{0}=0$, and (**b**) ${\Omega }^{{{\prime}}}/{\Gamma }_{0}=\Omega /{\Gamma }_{0}$.

Figure 8 demonstrates the evolution of dressed state’s population. According to 8(a), the dressed state $|\alpha \rangle$ has no population distribution, and the population are equally distributed in other three dressed states. In this case, the system operates as a three-level dressed atom, and the maximum value of normalized $DEM$ should be $\left({{\log}}_{2}3/{{\log}}_{2}4\right)=0.79$. This anticipation is in a good agreement of obtained results in Figs. 5a and 6a. In Fig. 8b, all the levels are populated, and the population distributed are almost equal in five dressed states. In this regards, the system acts as a five-level atom, and the maximum value of normalized $DEM$ should be equal to $\left({{\log}}_{2}5/2.32\right)=1$. This is also covering the obtained result of Figs. 5b and 6b.

Physically, existence of plasmonic nanostructure affects the transition $|3\rangle$ ($|4\rangle$) $\to |2\rangle$ that appears in parameter $\kappa$. In fact, the five-level atomic system has two $\mathrm{V}$-type transitions $|i\rangle \to |1\rangle$, and $|i\rangle \to |2\rangle$ ($i=3, 4$). The second transitions are coupled due to existence of plasmonic nanostructure that creates the parameter $\kappa$. These transitions may destroy the equality of the population distribution that leads in reduction of atom-photon entanglement as can be viewed in Figs. 7a and 8a. However, this may be balanced by the other laser field namely ${\Omega }^{{{\prime}}}$ as can be confirmed in Figs. 7b and 8b.

## Conclusion

The entanglement of a five-level atomic system and its spontaneous emission field is investigated with and without plasmonic nanostructure. For two linear laser fields, two different cases are examined. For turn off control laser field, the five-level system converts to a four-level one. In free space, the degree of created entanglement in five-level atomic system with its spontaneous emission is larger than the four-level atom. In the vicinity of the nanostructure, the atom-photon entanglement is affected by the distances of the atomic system from plasmonic nanostructure. The degree of entanglement depends on the distance of atom and the plasmonic nanostructure. Maximal atom-photon entanglement is obtained for a distance of $52 \; \text{nm}$ from the nanostructure. Because no coupling field drives the level $|2\rangle$, the only way to populate this level is spontaneously emitted photons from higher levels. Then, we are able to control the quantum interference using plasmonic nanostructure and therefore can control the spontaneous emission from level $|3\rangle$ and level $|4\rangle$ to level $|2\rangle$. By controlling the amount of population of level $|2\rangle$, we will be able to control the amount of normalized entanglement.

## Supplementary Information

Supplementary Information.
