# Supplementary material for: Controllable atom-photon entanglement via quantum interference near plasmonic nanostructure
Source: Sci Rep. 2022 Jan 13;12:677. doi: 10.1038/s41598-021-04641-6 (PMC8758766; doi:10.1038/s41598-021-04641-6)
Supplement: Supplementary file 1 — Supplementary Information. [file 41598_2021_4641_MOESM1_ESM.docx]

**Controllable atom-photon entanglement via quantum interference near plasmonic nanostructure**

Behzad Sangshekan^1*^, Mostafa Sahrai^1^, Seyyed Hossein Asadpour^2^, Jafar Poursamad Bonab^3^

^1^Faculty of Physics, University of Tabriz, Tabriz, Iran

^2^Young Researchers and Elite Club, Central Tehran Branch, Islamic Azad University, Tehran, Iran

^3^Department of Optical and Laser Engineering, University of Bonab, Bonab, Iran

^*^Email: [b.sangshekan@tabrizu.ac.ir](mailto:b.sangshekan@tabrizu.ac.ir)

**Supplementary note:**

Substituting equation (3) in equation (4) in the main text, the density matrix equations of motion in rotating frame are obtained as

|  | $\dot{\rho}_{11}=2\gamma^{''}\rho_{22}+2\gamma^{'}\left( \rho_{33}+\rho_{44} \right)+2\gamma_{51}\rho_{55}-\frac{1}{2}i\Omega\left( \rho_{13}+\rho_{14} \right)+\frac{1}{2}i\Omega\left( \rho_{31}+\rho_{41} \right)$,  $\dot{\rho}_{22}=2\gamma_{52}\rho_{55}-2\gamma^{''}\rho_{22}+2\gamma\left( \rho_{33}+\rho_{44} \right)+2\kappa\left( \rho_{34}+\rho_{43} \right)$,  $\dot{\rho}_{33}=2\gamma_{53}\rho_{55}-2\left( \gamma^{'}+\gamma\right)\rho_{33}-\kappa\left( \rho_{34}+\rho_{43} \right)+\frac{1}{2}i\Omega\left( \rho_{13}+\rho_{31} \right)-\frac{1}{2}i\Omega^{'}\left( \rho_{35}-\rho_{53} \right)$,  $\dot{\rho}_{44}=2\gamma_{54}\rho_{55}-2\left( \gamma^{'}+\gamma\right)\rho_{44}-\kappa\left( \rho_{34}+\rho_{43} \right)+\frac{1}{2}i\Omega\left( \rho_{14}-\rho_{41} \right)-\frac{1}{2}i\Omega^{'}\left( \rho_{45}-\rho_{54} \right)$,  $\dot{\rho}_{55}=-2\left( \gamma_{51}+\gamma_{52}+\gamma_{53}+\gamma_{54} \right)\rho_{55}+\frac{1}{2}i\Omega^{'}\left( \rho_{35}+\rho_{45}-\rho_{53}-\rho_{54} \right)$,  $\dot{\rho}_{21}=-\gamma^{''}\rho_{21}-\frac{1}{2}i\Omega\left( \rho_{34}+\rho_{35} \right)$,  $\dot{\rho}_{31}=-\left( i\delta+\left( \gamma^{'}+\gamma\right) \right)\rho_{31}-\kappa\rho_{41}+\frac{1}{2}i\Omega\left( \rho_{11}-\rho_{33}-\rho_{34} \right)+\frac{1}{2}i\Omega^{'}\rho_{51}$,  $\dot{\rho}_{32}=\frac{1}{2}i\Omega\rho_{12}-\left( i\delta+\left( \gamma^{''}+\gamma^{'}+\gamma\right) \right)\rho_{32}-\kappa\rho_{42}+\frac{1}{2}i\Omega^{'}\rho_{52}$,  $\dot{\rho}_{41}=-\left( i\delta+\left( \gamma^{'}+\gamma\right) \right)\rho_{41}-\kappa\rho_{31}+\frac{1}{2}i\Omega\left( \rho_{11}-\rho_{43}-\rho_{44} \right)+\frac{1}{2}i\Omega^{'}\rho_{51}$,  $\dot{\rho}_{42}=-\left( i\delta+\left( \gamma^{''}+\gamma^{'}+\gamma\right) \right)\rho_{42}-\kappa\rho_{32}+\frac{1}{2}i\Omega\rho_{12}+\frac{1}{2}i\Omega^{'}\rho_{52}$,  $\dot{\rho}_{43}=-2\left( \gamma^{'}+\gamma\right)\rho_{43}-\kappa\left( \rho_{33}+\rho_{44} \right)+\frac{1}{2}i\Omega\left( \rho_{13}-\rho_{41} \right),-\frac{1}{2}i\Omega^{'}\left( \rho_{45}-\rho_{53} \right)$,  $\dot{\rho}_{51}=-\left( i\left( \delta^{'}+\delta\right)+\left( \gamma_{51}+\gamma_{52}+\gamma_{53}+\gamma_{54} \right) \right)\rho_{51}+\frac{1}{2}i\Omega^{'}\left( \rho_{31}+\rho_{41} \right)-\frac{1}{2}i\Omega\left( \rho_{53}+\rho_{54} \right)$,  $\dot{\rho}_{52}=-\left( i\left( \delta^{'}+\delta\right)+\left( \gamma^{''}+\gamma_{51}+\gamma_{52}+\gamma_{53}+\gamma_{54} \right) \right)\rho_{52}+\frac{1}{2}i\Omega^{'}\left( \rho_{32}+\rho_{42} \right)$,  $\dot{\rho}_{53}=-\left( i\delta^{'}+\left( \gamma^{'}+\gamma+\gamma_{51}+\gamma_{52}+\gamma_{53}+\gamma_{54} \right) \right)\rho_{53}-\kappa\rho_{54}+\frac{1}{2}i\Omega^{'}\left( \rho_{33}+\rho_{43}-\rho_{55} \right)-\frac{1}{2}i\Omega\rho_{51}$,  $\dot{\rho}_{54}=-\left( i\delta^{'}+\left( \gamma^{'}+\gamma+\gamma_{51}+\gamma_{52}+\gamma_{53}+\gamma_{54} \right) \right)\rho_{54}-\kappa\rho_{53}+\frac{1}{2}i\Omega^{'}\left( \rho_{34}+\rho_{44}-\rho_{55} \right)-\frac{1}{2}i\Omega\rho_{51}$, | (1) |
| --- | --- | --- |
